# Discovery of Lijianmin-Chengkun Complexes and Their Oncological Application in Osseous and Intraarticular Lesions Around the Knee

**DOI:** 10.3389/fsurg.2021.691362

**Published:** 2021-12-03

**Authors:** Xianhao Shao, Jianmin Li, Ailin Zhang, Yuan Yao, Feifei Sun, Zhenzhong Li, Tao Liu, Haiqing Hou, Qiuyao Li, Zhenfeng Li, Xiaofei Gao, Qiang Yang, Yuchun Li, Ka Li, Kun Cheng

**Affiliations:** ^1^Department of Orthopedics, Qilu Hospital, Cheeloo College of Medicine, Shandong University, Jinan, China; ^2^Rehabilitation Units, University of Canberra Hospital, Bruce, ACT, Australia; ^3^Department of Radiography, Qilu Hospital, Cheeloo College of Medicine, Shandong University, Jinan, China; ^4^Department of Pathology, Qilu Hospital, Cheeloo College of Medicine, Shandong University, Jinan, China; ^5^Department of Anatomy and Neurobiology, School of Basic Medical Sciences, Cheeloo College of Medicine, Shandong University, Jinan, China; ^6^Department of Orthopedics, Qilu Hospital (Qingdao), Cheeloo College of Medicine, Shandong University, Qingdao, China; ^7^Department of Laboratory, Qilu Hospital (Qingdao), Cheeloo College of Medicine, Shandong University, Qingdao, China; ^8^Department of Pathology, Qilu Hospital (Qingdao), Cheeloo College of Medicine, Shandong University, Qingdao, China

**Keywords:** LC complex, epiphyseal and metaphyseal canal, tumor recurrence, tumor dissemination, giant cell tumor

## Abstract

**Objective:** This research aims to refresh the limited understanding about the canal and vascular structures within the epiphysis and metaphysis of the tibia and femur and their oncological significance.

**Methods:** This study was started with characterization of a novel structure using radiographs and anatomic dissections, followed by a descriptive clinical study with 55 participants to investigate the effects of tumors on this novel discovery and a retrospective cohort study with 82 participants to investigate whether the structure would be a risk factor for tumor recurrence after the curettage of giant cell tumor of bone.

**Results:** A new anatomical knee structure, the Lijianmin-Chengkun (LC) complex, was discovered in healthy adults, and its clinical implications were examined in this study. This new-found anatomical structure is composed of an epiphyseal and metaphyseal canal which surrounds a blood vessel, foramen, and foramen-covered synovium. All LC complexes showed similar radiographical, anatomical, and histological characteristics and were located within specific tibial and femoral intercondylar regions. These LC complexes seem to facilitate tumor residue and extension and may be a risk factor for tumor recurrence after curettage of femoral and tibial giant cell tumors (*P* = 0.031).

**Conclusion:** The LC complexes are related to local tumor recurrence and bidirectional tumor dissemination between intraosseous and intraarticular regions. These findings have opened up a new perspective and may provide new targets for intervention in malignant and aggressive tumors around the knee joint.

## Introduction

The knee joint has been subjected to extensive orthopedic research over the last century, mainly due to its complex anatomical structure and the fact that it is commonly affected in a variety of orthopedic diseases (1–3). The macroscopic anatomy and clinical significance of the tibial plateau, intercondylar fossa (ICF), and anterior and posterior cruciate ligaments (ACL & PCL) have been well-established (3–5). In recent years, the discovery of new knee joint-related anatomical structures has been rare (6).

The middle genicular artery (MGA) plays a pivotal role in supplying blood to the knee joint (7). Previous studies have focused on the middle genicular vein (MGV) (8), vena comitans of the MGA, and larger branches of the MGA (9, 10). However, there is a lack of research and analysis concerning the terminal branches or tributaries of the genicular vessels, and no information has been provided about how these vessels penetrate bone through fixed foramina and canals. Compared to the nutrient foramen and canal in the diaphysis (11, 12), the vasculature-canal structures that traverse the subchondral bone and epiphysis to the metaphysis of femur and tibia have not been investigated. In 2019, the network of transcortical capillaries (TCVs) in the mouse and human tibia was discovered, which prompted the investigation of the circulatory and channel system in cortical bone (13), suggesting that the intraosseous vasculature and canal are worthy of further study.

Our research idea originated in the orthopedic surgical practice; unexpected bleeding was found when we touched the ICF and tibial intercondylar region proximal to the center point during surgery. The observations inspired us to think about two questions. First, is it possible for certain small blood vessels to penetrate the bone through the tibial intercondylar region or the ICF within any relatively fixed, undiscovered canal structures? Second, what is the potential anatomical and clinical significance of this structure? With these questions, we have systemically studied these structures using radiographic, anatomical, and histological methodologies. This led to the discovery and characterization of tibial and femoral Lijianmin-Chengkun (LC) complexes which were composed of the foramen of the tibial intercondylar eminence (FTIE) or intercondylar fossa foramen (IFF), foramen-covered synovium, small blood vessels, and bony canal.

Giant cell tumor of bone (GCTB), the most common primary bone-related tumor around the knee in young and middle-aged Asian adults, has been well-investigated (1). Subchondral destruction and filling material may affect functional outcomes after curettage for GCTB (1, 14). However, the potential “breakpoint” on the subchondral bone plate and the related negative oncological prognosis was not mentioned previously. Upon the discovery of the LC complexes in the present study, we conduct a descriptive study to investigate the general oncological implications of these new identified structures. Based on this, we developed more specific studies on whether the LC complex would increase and accelerate the local recurrence of GCTB in the subchondral area and analyzed the data statistically. The data of the present study not only identified new vasculature-canal structures in the distal femur and proximal tibia but also provided the clinical evidence that the LC complexes can contribute to the recurrence and spread of knee joint tumors.

## Materials and Methods

All studies were conducted in Qilu Hospital, Qilu Hospital (Qingdao) and Department of Anatomy and Neurobiology of Shandong University with the approval of the institutional ethics committee. For a full description of the patients and methods, please see the Supplementary Material.

### Radiographic Observation and Measurement

The occurrence probability, number, and diameters of the FTIE and IFF were measured using computed tomography (CT). Magnetic resonance imaging (MRI) was used to observe the components of the LC complex and measure certain distances in relation to the FTIE or IFF (Table 1). We calculated DA/(DA+DP) and DM/(DM+DL) to determine the location of the FTIE and IFF along anteroposterior and mediolateral lines, respectively.

**Table 1:** Definition of the measured parameters indicating the location of FTIE and IFF (mm).

**Foramen**	**Definition**	**Explanation**
FTIE	DA	The distance between FTIE and anterior margin of tibia plateau
FTIE	DP	The distance between FTIE and the intersection point of DA extending line and the sagittal line on which the posterior margin of tibia plateau lied
FTIE	DM or DL	The distance between the projection of FTIE on the coronary baseline which created by connecting the medial and lateral margin of tibia plateau and the medial or lateral margin of tibia plateau, respectively
IFF	DA or DP	The distance between IFF and anterior or posterior margin of ICF, respectively
IFF	DM or DL	The distance between IFF and medial margin of medial femoral condyle(MFC) or lateral margin of lateral femoral condyle(LFC), respectively
FTIE& IFF	DACL	The distance between FTIE or IFF and the posterior margin of tibial or femoral ACL insertion, respectively
FTIE&IFF	DPCL	The distance between FTIE or IFF and the anterior margin of tibial or femoral PCL insertion, respectively

*More specific explanations for the measurements were shown in Supplementary Figure 1*.

*FTIE, foramen of tibial intercondylar eminence; IFF, intercondylar fossa foramen; DA, distance to anterior margin; DP, distance to posterior margin; DM, distance to medial margin; DL, distance to lateral margin; DACL, distance to anterior cruciate ligament; DPCL, distance to posterior cruciate ligament; MFC, medial femoral condyle; LFC, lateral femoral condyle; ICF, intercondylar fossa*.

To facilitate the analysis of the images, we established a nine-zone model based on 3D-CT reconstruction by dividing the specified femoral or tibial intercondylar area into nine equal-sized zones. The edges of the nine tibial and femoral zones were chosen (Table 2), using the model we counted the number of FTIE and IFF in each zone. All the observations were performed by two experienced researchers and all the measurements were reviewed in three planes (axial, coronal, and sagittal) to ensure the authenticity of FTIE or IFF.

**Table 2:** Definition of the edges in Nine-Zone model.

**Foramen**	**Edge**	**Explanation**
FTIE	Medial edge	Lateral sagittal tangent of medial intercondylar tubercle
FTIE	Lateral edge	Medial sagittal tangent of lateral intercondylar tubercle
FTIE	Anterior edge	A coronal line in front of the center of the tibial plateau, located with a distance of 15% of maximum length on anteroposterior line of tibia plateau from the center of the tibial plateau.
FTIE	Posterior edge	A coronal line behind the center of the tibial plateau, located with a distance of 15% of maximum length on anteroposterior line of tibia plateau from the center of the tibial plateau.
IFF	Medial edge	Lateral sagittal tangent of MFC
IFF	Lateral edge	Medial sagittal tangent of LFC
IFF	Anterior edge	The coronary line on which anterior margin of ICF situated
IFF	Posterior edge	The coronary line on which posterior margin of ICF situated

*FTIE, foramen of tibial intercondylar eminence; IFF, intercondylar fossa foramen; MFC, medial femoral condyle; LFC, lateral femoral condyle; ICF, intercondylar fossa*.

### Dissection Observation and Measurement

Sixty healthy adult knees from cadavers or amputated lower extremities and 14 knees from patients with tumors were dissected. First, we exposed the knee joint to find the MGA and MGV. When dissecting along the MGA branches or MGV tributaries to the ICF or tibial intercondylar region, we clearly visualized the LC complex. Next, we measured the DA, DP, DM, and DL to identify the FTIE and IFF in specimens using the same method as described in the radiographic measurement. The number of FTIEs and IFFs in each zone was also counted, and the margins of the tumor on the joint surface of the tibial plateau or ICF were measured in tumor specimens. Finally, we dissected the intercondylar region to expose the intraosseous components of the LC complex. We examined the anatomic features of each component in the LC complex and focused on discerning any involvement between the LC complex and tumor growth.

### Descriptive Clinical Study

Fifty-five patients (28 male, 27 female), with an average age of 33.26 ± 10.54 years (18–69 years old) who underwent surgery, adjuvant therapy, biopsy, or telemedicine in our hospitals were enrolled in the descriptive clinical study from May 2017 to October 2021. Tumor involvement in LC complexes was observed using radiographic, intraoperative, and pathological methodologies. The specific lesions included 20 osteosarcomas, 22 GCTBs, five diffused giant cell tumors of the tendon sheath, four chondrosarcomas, three metastatic tumors, and one Ewing sarcoma.

The exclusion criteria for this study included: (1) patients with congenital malformations of knee joint; (2) patients with inadequate radiographs of the tumor involved LC complexes; and (3) patients who did not consent to the inclusion of their private clinical information in this study.

### Retrospective Clinical Study

**Figure 1:**
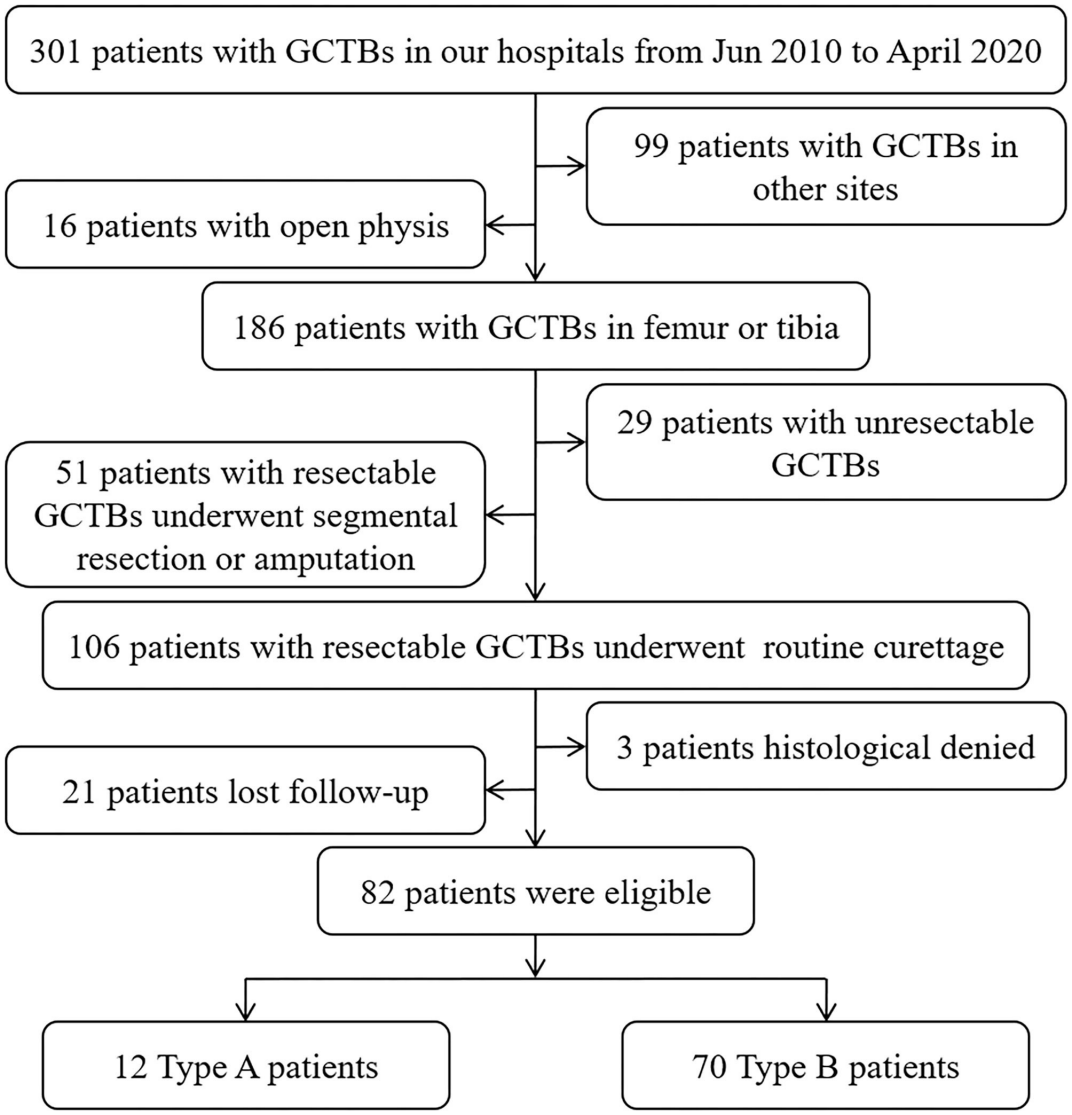
Flow chart illustrating the inclusion and exclusion criteria of the retrospective study.

To analyze whether GCTB involvement in the subchondral LC complex would be a potential risk factor for tumor recurrence after curettage, a retrospective cohort including 82 patients (42 male, 40 female) with average age 35.88 ± 11.09 years old (18–63 years) who suffered resectable GCTB and underwent curettage of the femur or tibia from June 2010 to April 2020 was established in this study (Figure 1).

We categorized the eligible patients into two types to evaluate the discrepancy in the recurrence rate caused by subchondral invasion of the LC complex. Type A patients were those with a tumorous lesion that touched the LC canals in the previously described subchondral area (1); sometimes, the FTIE or IFF was also involved. Type B patients had GCTBs in other intraosseous regions. A qualified Type A or Type B patient was identified based on the radiographic manifestation on preoperative CT and MRI, and intraoperative observation. The clinical and imaging information of these patients with GCTB was reviewed retrospectively.

### MicroCT Imaging

Quantitative multiphase bone images were acquired using a Quantum GX microCT scanner (PerkinElmer, Inc, Waltham, MA). The samples of normal femoral and tibial LC complexes were dissected and cut to appropriate size for microCT scanning. Once a qualified sample was achieved (verified by the scanner), the scanner bed was translated longitudinally to align the sample within the center of the field of view. The scanner's complementary metaloxide-semiconductor X-ray flat-panel detector was set to allow image acquisition with a temporal resolution of 16 ms and an X-ray tube voltage of 90 kV and current of 88 μA. Raw projection images were processed using a proprietary algorithm for bone and then reconstructed using a filtered back-projection algorithm on a dedicated graphics processing unit.

Reconstructed volumes were loaded into Analyze 12 software (Analyze Direct, Mayo Clinic). Images were then binarized using uniform thresholding. The uniform threshold value was defined as the value which could judge the cortical bone (white) from other tissues (green) in each sample. All microCT images were processed by 2 observers blinded to the other results in this investigation.

### Hematoxylin-Eosin (HE) Staining

Routine HE staining was performed as previously described (15). Post-HE staining was performed on the mounted specimen slides, and images were taken using an Olympus IX-70 microscope (Tokyo, Japan).

### Angiography

Normal human angiography was conducted on a healthy volunteer (male, 41-year-old) in the supine position using an angiography X-ray machine (Artis Zee floor, Germany) after injecting 30 mL contrast agent (iohexol 300 mg/mL) into the popliteal artery. We then observed the terminal arteries within the tibial or femoral intercondylar regions.

### Statistics Analysis

Data were analyzed using SPSS 21.0 and MATLAB 2016b. Student's *t*-test was used to analyze the statistical differences in the location of FTIE and IFF in different samples. The Kolmogorov–Smirnov (K-S) test was used to analyze the distribution of FTIE and IFF locations. A Kaplan–Meier curve was created to analyze whether GCTB involvement in the subchondral LC complex was a risk factor for local recurrence. Statistical significance was set at *P* < 0.05. *P* < 0.01 was considered highly statistically significant.

## Results

### Findings in Healthy Adult LC Complexes

**Figure 2:**
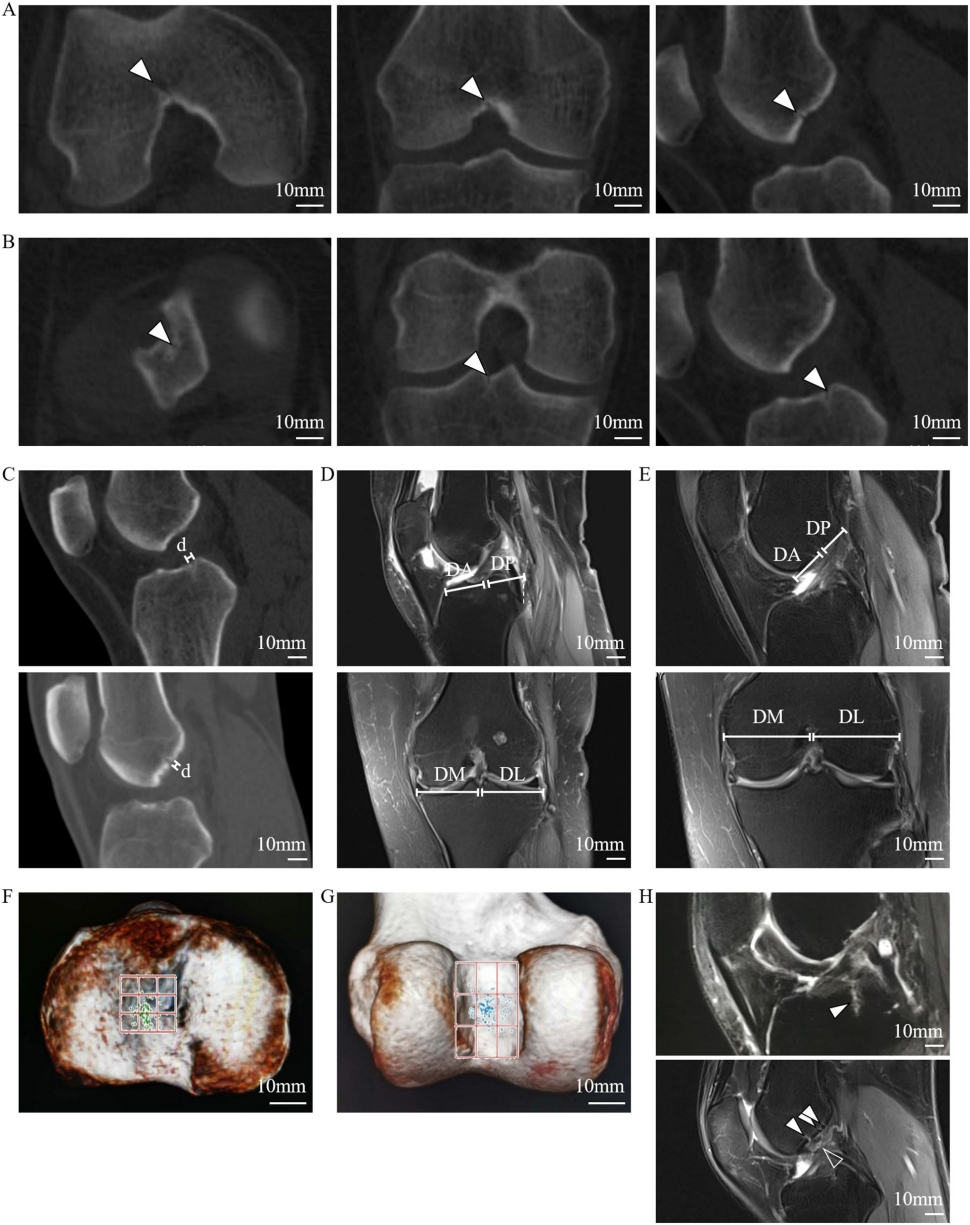
Radiographic features of the FTIE, IFFs, and enclosed tissues of the LC complex. **(A)** IFF (arrowheads) shown on 1-mm-thick reconstructed sagittal and coronal CT images. **(B)** FTIE (arrowheads) shown on 1-mm-thick reconstructed sagittal and coronal CT images. **(C)** Radiographic measurement of diameters of the FTIE and IFF shown as “d.” **(D,E)** Radiographic measurement of the DA, DP, DM, and DL of the FTIE **(D)** and IFF **(E)**. **(F)** The distribution of FTIEs in the nine-zone model. **(G)** The distribution of IFFs in the nine-zone model. **(H)** The soft tissues (arrowheads) within the tibial and femoral LC complexes shown on MRI that appear to connect to the branches of the MGA or tributaries of the MGV (empty arrowhead).

Using 1-mm-thick CT images, the reconstructed sagittal and coronal images (*n* = 200) showed the FTIE and IFF as low-density structures in the tibia and femur. Only one FTIE (mean diameter of 1.28 ± 0.16 mm, *n* = 200), which connects to a low-density canal extending to the tibial spongy bone, was found near the tibial intercondylar eminence (TIE). Two to four IFFs (mean diameter of 1.21 ± 0.13 mm, *n* = 588) connecting to low-density canals extending to the femoral spongy bone were found in the ICF (Figures 2A–C).

The localization of 100 FTIEs and 225 IFFs was examined using 142 MRIs or contrast-enhanced MRIs with the measurement of DA, DP, DM, and DL (Figures 2D,E). DA/(DA+DP) was 55.09% ± 4.60% (*n* = 100) and 49.43% ± 9.31% (*n* = 225) for FTIE and IFF, respectively. DM/(DM+DL) was 48.88% ± 2.89% (*n* = 100) and 50.38% ± 3.87% (*n* = 225) for FTIE and IFF, respectively (Table 3). In fifty 3D-CT reconstructed images, 78.0% of FTIEs were located in Zone 5 or Zone 8, while 68.0% of IFFs were located in Zone 5 (Figures 2F,G). On MRI, we observed that the bony canals connecting to the FTIEs or IFFs contained strip structures with vascular features. Some images clearly showed that these potential vasculatures may have a certain continuity with the middle genicular vessels (Figure 2H).

**Table 3:** Relative location of IFF and FTIE in MRI measurements.

	**Mean location on**	**Mean location on**	**Located region on**	**Located region on**
	**anteroposterior line**	**mediolateral line**	**anteroposterior line**	**mediolateral line**
IFF	49.43% ± 9.31%	50.38% ± 3.87%	31.2–67.7%	42.8–58.0%
FTIE	55.09% ± 4.60%	48.88% ± 2.89%	46.1–64.1%	43.2–54.5%

*Located regions on anteroposterior and mediolateral lines were calculated by the 95% confidence interval (CI) with K-S test. FTIE, foramen of tibial intercondylar eminence; IFF, intercondylar fossa foramen*.

**Figure 3:**
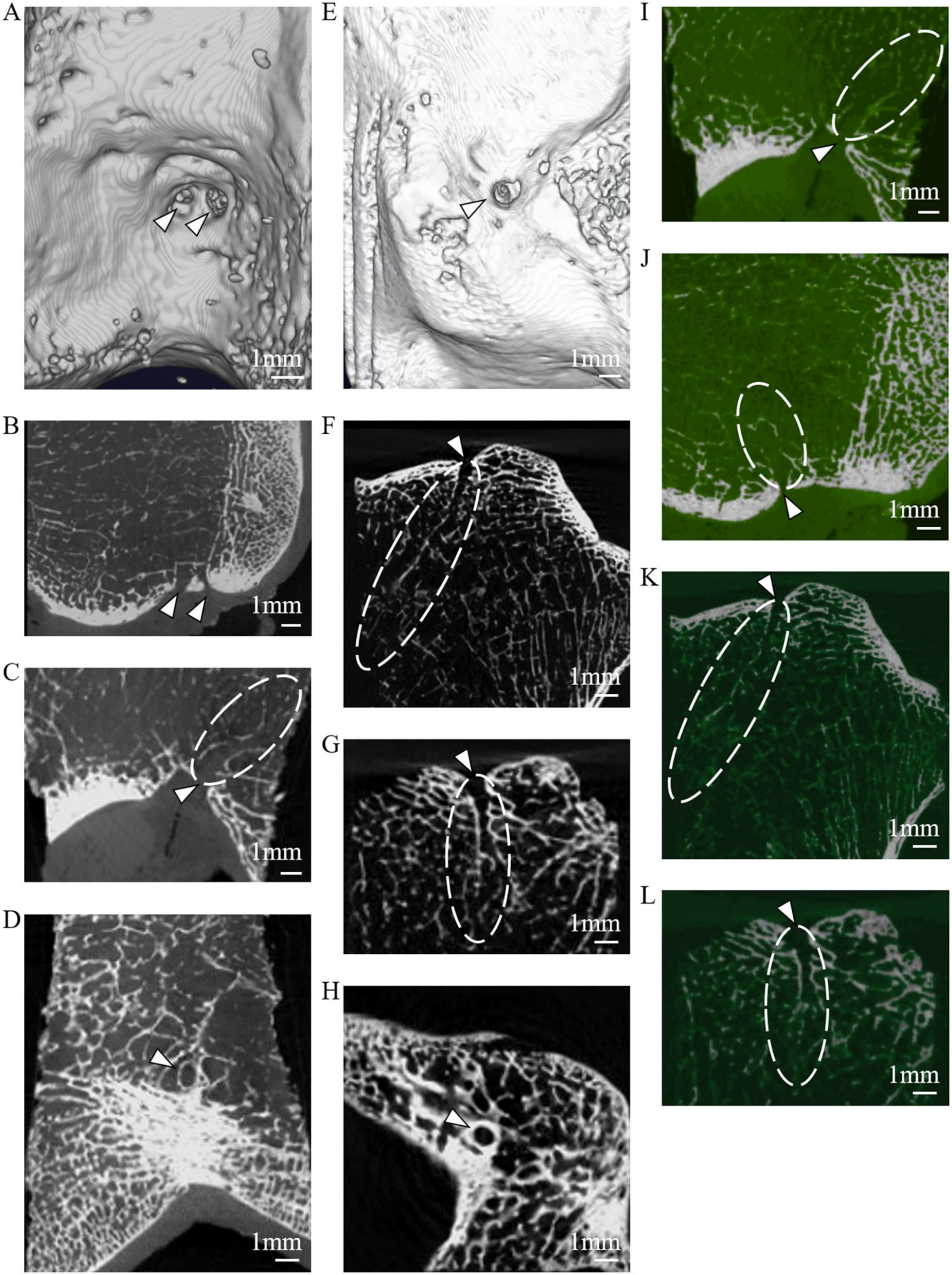
The microarchitectural characterization of the LC complex under microCT. **(A)** IFFs (arrowheads) shown on reconstructed 3D microCT images. **(B)** IFFs (arrowheads) shown on reconstructed coronal microCT images. **(C)** IFF (arrowhead) and femoral LC canal (dashed line) shown on reconstructed sagittal microCT images. **(D)** IFF (arrowhead) shown on axial microCT images. **(E)** FTIE (arrowhead) shown on reconstructed 3D microCT images. **(F)** FTIE (arrowhead) and tibial LC canal (dashed line) shown on reconstructed sagittal microCT images. **(G)** FTIE (arrowhead) and tibial LC canal (dashed line) shown on reconstructed coronal microCT images. **(H)** FTIE (arrowhead) shown on axial microCT images. **(I)** IFF (arrowhead) and femoral LC canal (dashed line) shown on reconstructed threshold-binarized sagittal microCT images. **(J)** IFF (arrowhead) and femoral LC canal (dashed line) shown on reconstructed threshold-binarized coronal microCT images. **(K)** FTIE (arrowhead) and tibial LC canal (dashed line) shown on reconstructed threshold-binarized sagittal microCT images. **(L)** FTIE (arrowhead) and tibial LC canal (dashed line) shown on reconstructed threshold-binarized coronal microCT images.

To further investigate the osseous features of LC complexes, the microarchitectures of femoral and tibial specimens were sequentially characterized under microCT. The scanned images of microCT distinctly showed the FTIE and IFF on the articular facet, moreover, the canals connecting to the FTIEs or IFFs breached the cortical subchondral bone plate and extended to the region of cancellous bone in all samples (Figures 3A–H). On reconstructed sagittal and coronal microCT images, the walls of these canals appeared bony features (Figures 3B,C,F,G). In the threshold-binarized microCT images which was processed by Analyze 12 software, it was found that the subchondral bone plate of distal femur and proximal tibia was displayed as continuous cortical bone except for the FTIE and IFF which showed different density and appeared as the “breakpoints” (Figures 3I–L). The canals connecting to the FTIEs or IFFs could traverse the epiphysis and extend to the further metaphysis (Figures 3I–L).

**Figure 4:**
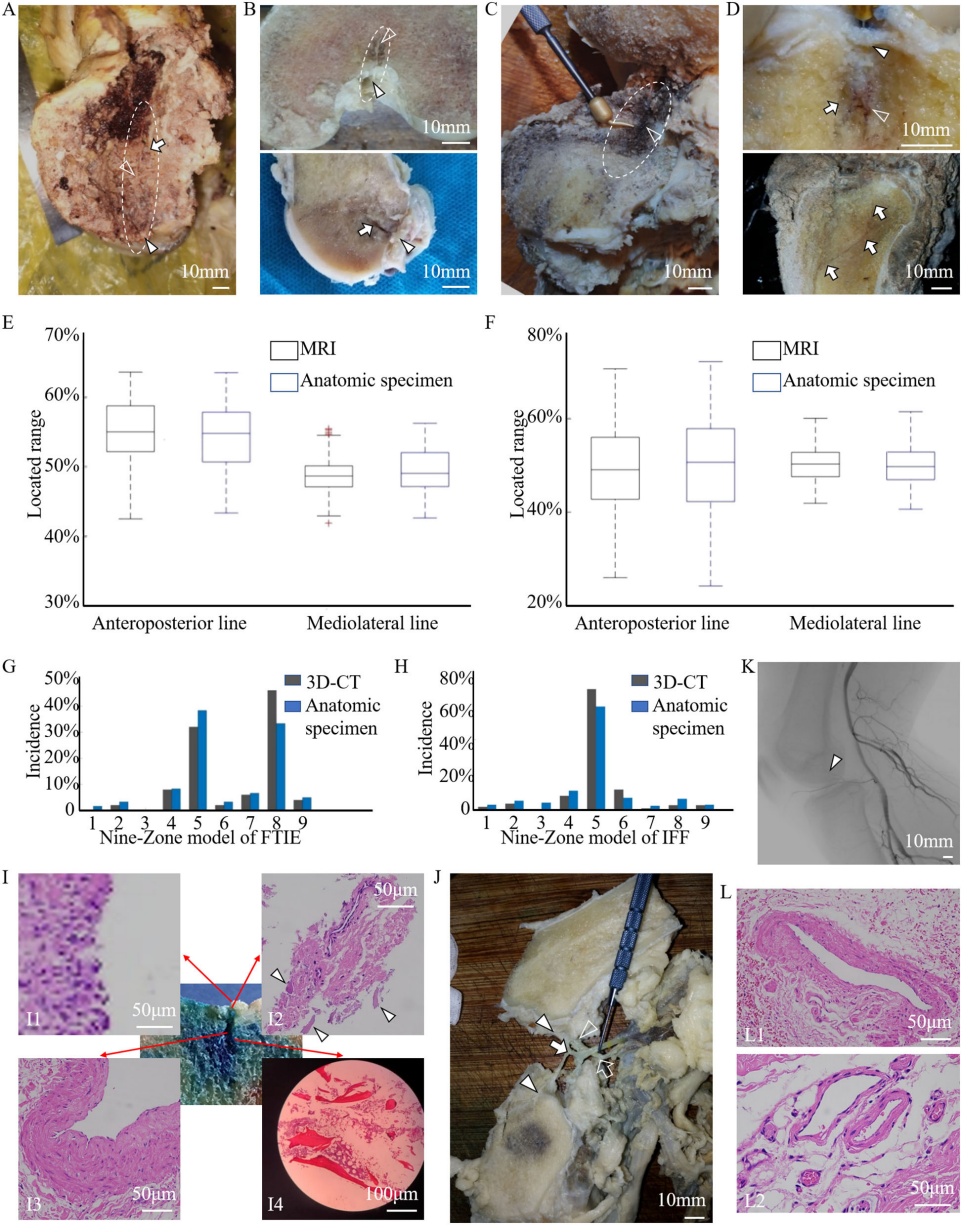
The anatomic, histologic, and angiographic features of the LC complex. **(A,B)** The foramina-covered synovium (arrowheads), LC vessels (empty arrowheads), and wall of bony canals (arrows) of the femoral LC complex (dashed line) on the gross anatomical view **(A)**, and coronal and sagittal sections **(B)** of the specimens. **(C,D)** The foramina-covered synovium (arrowhead), LC vessels (empty arrowheads), and wall of bony canals (arrows) of the tibial LC complex (dashed line) on the gross anatomical view **(C)**, and coronal and sagittal sections **(D)** of the specimens. **(E,F)** The location of FTIE **(E)** or IFF **(F)** determined on enrolled MR images (black boxes), and by direct measurements in anatomic specimens (blue boxes) of healthy donors were expressed as the percentage of DA/(DA+DP) and DM/(DM+DL) to conduct the quantitative comparison. Boxplots show averaged and minimum/maximum location values. No statistical difference is found along the anteroposterior (tibia: *P* = 0.212, femur: *P* = 0.421) and mediolateral (tibia: *P* = 0.379, femur: *P* = 0.184) lines. **(G,H)** The distributions of FTIE **(G)** and IFF **(H)** in the nine-zone model in the 3D-CT reconstruction (black columns) were consistent with that in the anatomic specimen (blue columns). **(I)** The histological characterization of the main components of the tibial LC complex (methylene blue stained area) using HE staining, including the foramina-covered synovium (I1), foramina walls (arrowheads) (I2), enclosed vessel (I3), and cancellous bony wall of the canal (I4). **(J)** The gross anatomical dissection showing the branches of the MGA (empty arrow) penetrating the intercondylar fossa (empty arrowhead) and the tributaries of the MGV (arrow) penetrating both the intercondylar fossa and tibial intercondylar eminence (arrowheads). **(K)** The angiographic image of the human body shows the existence of small arteries in the intercondylar fossa (arrowhead). **(L)** HE images showing venous features in the tibial LC vessel (L1), and venous and arterial features in the femoral LC vessels (L2).

The dissection results of 60 adult knee specimens showed consistent findings with the radiographic observations of 2–4 femoral LC complexes and one tibial LC complex on articular facets. Each LC complex contained an IFF or an FTIE. The femoral and tibial LC complexes originate from the intraarticular region, pass through the articular cartilage and subchondral bone, and penetrate into the cancellous bone to form bony canals in the intercondylar epiphysis and metaphysis of the distal femur and proximal tibia, respectively. A vasculature-like soft tissue within each LC bony canal and the foramina covering situated between the IFF (FTIE) and articular cavity were observed in all femoral and tibial LC complexes (Figures 4A–D).

Using FTIE and IFF as canonical landmarks for the LC complex, we determined their location information using dissections (Table 4). When comparing the measurement results of the anatomic dissections with those made using the MRIs, we found no statistical differences in either the anteroposterior or mediolateral lines (Figures 4E,F). In the nine-zone model, 76.3% of the FTIEs were located in Zone 5 or Zone 8 and 55.0% of the IFFs were located in Zone 5 (Figures 4G,H).

**Table 4:** Relative location of IFF and FTIE in normal adult anatomic specimens.

	**Mean location on**	**Mean location on**	**Located region on**	**Located region on**
	**anteroposterior line**	**mediolateral line**	**anteroposterior line**	**mediolateral line**
IFF	50.26% ± 10.75%	49.81% ± 4.48%	29.2–71.3%	41.0–58.6%
FTIE	54.11% ± 4.91%	49.35% ± 3.39%	44.5–63.7%	42.7–56.0%

*Located regions on anteroposterior and mediolateral lines were calculated by the 95%CI with K-S test. FTIE, foramen of tibial intercondylar eminence; IFF, intercondylar fossa foramen. Total 60 FTIEs and 175 IFFs were observed in the 60 normal adult knees*.

HE staining showed that both FTIE and IFF contained osseous tissue. Bone lamellae and bone marrow cells were observed in the walls of the epiphyseal and metaphyseal canals. The vasculature, which was surrounded by peripheral connective tissues within the canal, had adventitia, media, and intima. The foramina-covered tissues appeared to be typical synoviocytes and loose connective tissues (Figure 4I).

During the dissection, we observed that the tibial LC vessel was directly connected to the MGV tributaries, while all femoral LC vessels were directly connected to the MGA branches or MGV tributaries (Figure 4J), which was also supported by angiography (Figure 4K). To ensure the identity of the blood vessels, we performed HE staining of 23 femoral LC vessels and 10 tibial LC vessels. The results showed venous features in 12 femoral and 10 tibial LC vessels, and arterial features in another 11 femoral vessels, but not in tibial LC vessels (Figure 4L).

### LC Complex Involvement in Knee-Related Tumors

**Figure 5:**
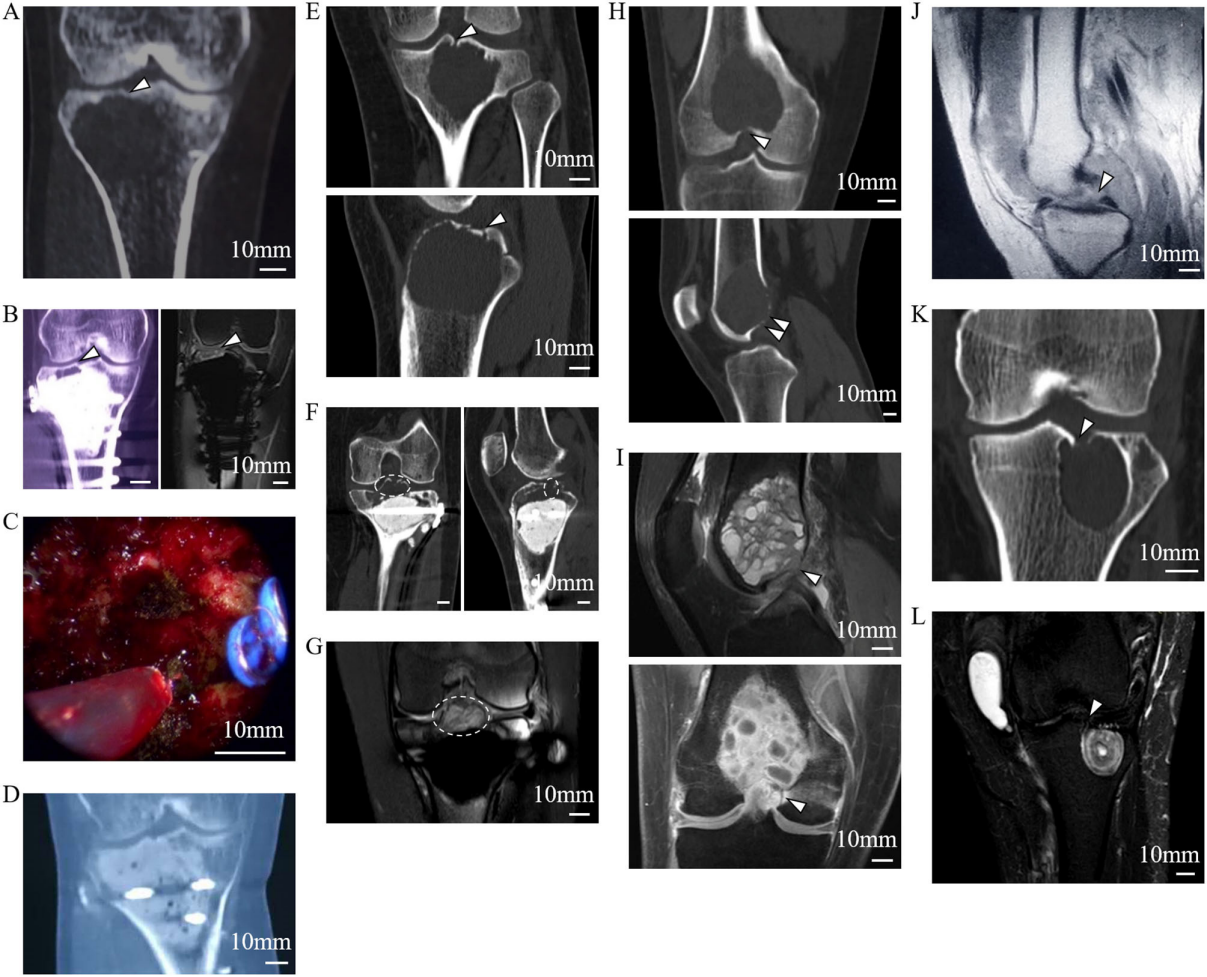
Tumor residue and extension in the LC complex on radiographic and intraoperative images. Images **(A–D)** from a 46-year-old female patient with recurrent GCTB. **(A)** Suspicious tumor residue shown in the tibial subchondral LC complex (arrowhead) in the first recurrence on CT. **(B)** Obvious GCTB recurrence shown in the tibial subchondral LC complex (arrowheads) on CT and MRI in the second recurrence. **(C)** LC complex ablation under arthroscopy. **(D)** No recurrence was observed for 24 months after LC complex ablation. Images **(E–G)** from a 20-year-old female patient with GCTB disseminating from the tibia to the articular cavity via the tibial LC complex. **(E)** Tumor invasion of the tibial LC complex (arrowheads) on the CT coronal and sagittal reconstruction before first operation. **(F,G)** The tumor recurred 13 months after curettage and extended into the articular cavity via the tibial LC complex (dash lines), as seen on CT **(F)** and MRI **(G)**. Images **(H,I)** from a 24-year-old male patient with GCTB extension from the femur to the articular cavity via the femoral LC complexes (arrowheads) on CT **(H)** and MRI **(I)**; the GCTB was confirmed following needle biopsy. Images **(J–L)** from a 52-year-old woman with recurrent diffused giant cell tumor of the tendon sheath; the intraarticular lesion disseminated to the tibia via the tibial LC complex. **(J)** D-TGCTS located above the tibial intercondylar region (arrowhead) on the MRI before synovectomy. **(K)** Osteolytic lesion occurred in the tibial LC complex (arrowhead) and intraosseous lesion shown on CT at 6 years after the synovectomy. **(L)** Tumor spread via the tibial LC complex (arrowhead) without damaging the peripheral cartilage on MRI.

In the descriptive clinical study of 55 patients, 17 patients had potential tumor residue, which was confirmed through medical history, radiography, and intraoperative observations of their LC complexes, suffered tumor recurrence (Figures 5A,B). When GCTB lesions appeared in the LC complex at the subchondral or articular level, the local recurrence rate was quite high in the ten patients who underwent curettage (5/10, 50.0%). However, three patients who experienced recurrent GCTB underwent LC complex ablation (Figure 5C) in combination with traditional curettage, and recurrence was not found after ablation during the follow-up period from nine to 32 months (Figure 5D). These results suggest a potential correlation between the subchondral LC complex and GCTB recurrence.

The bidirectional tumor extension between intraosseous and intraarticular regions via femoral or tibial LC complexes was another typical manifestation during our observation and occurred in 21 of the 55 patients. The GCTB could penetrate into the joint cavity through the tibial LC complex during recurrence (Figures 5E–G). In addition, intraarticular tumor extension was also seen in primary GCTB, which spread via femoral LC complexes (Figures 5H,I). Diffused giant cell tumor of the tendon sheath was also able to invade into the tibial metaphysis from the intraarticular region, suggesting the juxta-articular tumorous dissemination via the LC complex seemed to be bidirectional (Figures 5J–L).

**Figure 6:**
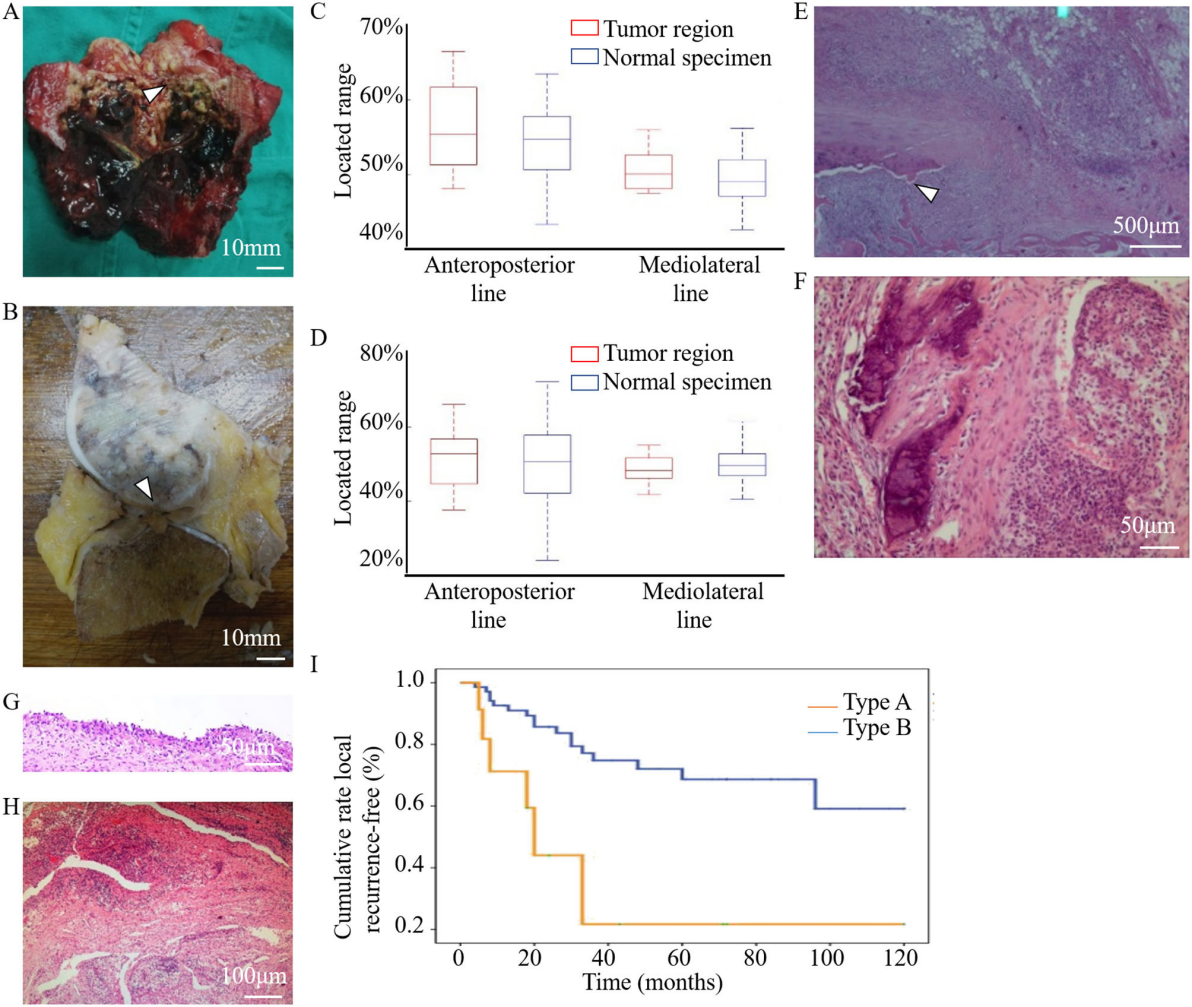
Anatomical and pathological images showed the involvement of the LC complex in tumor and the cumulative recurrence-free survival was shown in the Kaplan–Meier curve. **(A)** Tibial LC complex invaded by malignant GCTB (arrowhead) in anatomic specimen. **(B)** Femoral LC complex infiltrated by osteosarcoma (arrowhead) in anatomic specimen. **(C,D)** The quantitative comparison of tumor-involved regions from tumor patients (*n* = 14, eight tibias **(C)** and six femurs **(D)**) and the region of the normal LC complex from healthy donors (*n* = 60) was analyzed using boxplots. No statistical difference was found along the anteroposterior (tibia: *P* = 0.451, femur: *P* = 0.714) or mediolateral (tibia: *P* = 0.353, femur: *P* = 0.463) lines. Images **(E–H)** were obtained following HE staining to illustrate the pathological changes of LC complex. **(E)** Tumor extension via the femoral LC complex (arrowhead) without damaging the articular cartilage in the vicinity. **(F–H)** Pathological changes in the foramina-covered synovium of the LC complex. **(F)** Synovium with tumor infiltration. **(G)** Synovial chronic inflammation. **(H)** Synovial chronic inflammation and interstitial hyperemia. **(I)** Kaplan–Meier curve of the cumulative rate without local recurrence by tumor location. With Type B patients as reference, the HR (95% CI) of local recurrence was 2.794 (1.097–7.113) in Type A patients, *P* = 0.031.

Fourteen knees with tumors from patients who had undergone segmental resection or amputation were dissected for anatomical and pathological examinations. Tumor invasion of the LC complex was explicitly seen in the anatomic investigation (Figures 6A,B). There was no statistically significant difference between these tumor-involved area and the region of the normal LC complex on the articular surface (Table 5) (Figures 6C,D).

**Table 5:** Located region of tumors in femur and tibia.

	**Mean location on**	**Mean location on**	**Located region on**	**Located region on**
	**anteroposterior line**	**mediolateral line**	**anteroposterior line**	**mediolateral line**
Femur	51.51% ± 9.07%	48.64% ± 4.18%	33.7–69.3%	40.4–56.8%
Tibia	56.40% ± 6.76%	50.71% ± 3.16%	43.2–69.6%	44.5–56.9%

*Mean location on anteroposterior or mediolateral line was equal to the average value of the region with tumor involvement on articular facet. The overall located regions on anteroposterior and mediolateral lines were calculated by the 95%CI with K-S test*.

Microscopic observation showed that osteosarcoma cells only spread through the canal of the femoral LC complex without obvious destruction of the peripheral articular cartilage (Figure 6E). Pathological changes in the foramina-covered synovium in the LC complex were observed in all 14 specimens (14/14, 100%). Tumor infiltration and acute or chronic inflammation were the two most common pathological findings, with an occurrence of 50% (7/14) and 42.9% (6/14), respectively. Other pathological changes, such as degeneration, reactive hyperplasia, hyaline changes, and hyperemia, were also observed (Figures 6F–H).

In both the tibia and femur, an increased recurrence rate and shortened recurrence period were observed in Type A patients. Univariate analysis revealed that local recurrence was significantly associated with subchondral LC complex involvement after GCTB curettage (Figure 6I) (HR: 2.794, 95%CI: 1.097–7.113, *P* = 0.031).

## Discussion

In this study, we discovered and named a novel anatomical composite structure, the LC complex, and demonstrated its components. Both the FTIE and IFF are “openings” of the LC complex on the articular surface. They are inherent structures in the human body with relatively fixed locations and can be used as a landmark for locating the LC complex. Usually, there is one FTIE and two-four IFFs, which may be accompanied by accessory foramina (Supplementary Figure 2). However, no anatomical structure was found within the accessory foramina, suggesting its limited anatomical significance. In a previous study on rat tibia, the authors described the FTIE with its covering synovium and location adjacent to the ACL (16). In their research, they focused more on researching the passage of nerve fibers in the epiphysis of rats. To the best of our knowledge, there has been no systematic study on IFF in any animals and human beings. Our measurements demonstrate that the FTIE is usually located within the vicinity of the TIE, the IFF tends to be situated in the central region of the ICF, and there are certain distances from these foramina to the attachments of the ACL and PCL (Supplementary Figure 4).These results are supported by the data that have shown the length, shape, and location of the ACL and PCL at their tibial and femoral insertion (17, 18).The geometric measurement values for the FTIE and IFF are consistent between our radiographic and anatomic measurements; hence, we consider that FTIE and IFF are distributed in a relatively constant manner in certain regions of the tibial plateau and ICF, respectively. To better understanding the microstructure of LC complexes, we carried out the microCT scanning and data analysis. From the microCT images, we discovered that FTIE and IFF not only are foramina on the articular facet, but also connect to the canals that breach the subchondral bone plate and extend to the deep spongy bone area (Figure 3). This new discovery deepens our understanding on anatomical characteristics of LC complexes. The existence of the bony canals may provide the potential space which facilitates the tumor recurrence and dissemination. The measurement and microstructural identification of LC complexes provide an anatomical background for clinical studies and therapy, such as the accurate ablation of this structure.

We also determined the origin of the small blood vessels within the LC complex. Previous studies mainly focused on the blood supply, bifurcation, and common trunk of the MGA, rather than their entry into the bone (7–10). Some scholars believe that the branches of the MGA entering the ICF are random and do not pay much attention to the inherent foramen of entrance (7–10). In addition, histological verification has rarely been conducted on small vessels that penetrate the TIE (9). To our knowledge, no study has systematically described the terminal tributaries of the MGV. Our findings may add to the knowledge regarding the anatomy of these small vessels.

However, the proportion of arterial and venous blood flow in the metaphysis via LC vessels and physiopathological functions, such as transport of inflammatory cells or other molecules, warrants further investigation. The existence of this intraosseous canal assists peripheral capillaries to enter the vein within LC complexes. However, it may also increase the potential risk of pathogenic factor invasion. The foramen-covered synovium is deemed to be a physical and biological barrier for the LC complex because of its anatomic location and histological characteristics.

The results of our clinical investigations confirmed the oncological significance of LC complexes. Regarding patients with GCTB invasion of the subchondral LC complex, we observed a higher recurrence rate and shorter relapse period compared to those with GCTB lesions in other femoral or tibial regions after curettage. Several issues may be related to these results. First of all, it is difficult to conduct the thorough tumor curettage in the subchondral bone layer using a high-speed burr and electrocautery, which are usually selected to achieve an extensively safe margin of the tumor cavity in cancellous and cortical bone. Moreover, since several studies have demonstrated that the destruction of subchondral bone and cement packing will lead to poor functional outcomes after curettage, the protection or grafting of subchondral bone, and the diminished PMMA plugging in the subchondral area have been prompted (1, 19). In this situation, the subchondral area often receives limited oncological treatment and circumscribed curettage margins. Here, due to the neglect and inadequate treatment of this newly discovered “breakpoint” on the “continuous” subchondral bone plate, potential GCTB residues may be hidden in the subchondral LC complex, leading to frequent recurrence. The endoscopic ablation of subchondral LC complex, which achieved optimistic oncological outcomes in this study (Figure 5C), may therefore be recommended after strict clinical verification in the future.

According to our intuitive observation, tumor cells also undergo bidirectional propagation via the “bone-LC complex-articular cavity.” In many cases, tumors extend through the LC complex without damaging the peripheral cartilage and subchondral bone. Due to the vulnerability of the LC complex to juxta-articular tumor extension, we speculate that more segmental resection for aggressive tumors and extraarticular resection for malignant tumors may be performed in relevant cases. The pathological changes in the synovium of the LC complex occurred in all 14 tumor specimens. Therefore, we consider the foramen-covered synovium an indicator of tumor invasion.

Our study had several limitations. First, some disadvantages, such as unclear imaging of the foramina and difficulties in the measurement of specimens, emerged during our actual operations for geometric measurement of the FTIE and IFF. Therefore, we judged our data as a rough and primary measurement for the location of the LC complex, and expect that more precise and credible data should be available via modified methods in future studies. In addition, it is inevitable to exclude some patients with uncertain tumorous involvement in subchondral LC complexes because these structures are too small to be captured on each radiograph; thus, a bias may occur. Multivariate analysis for other potential risk factors, such as age, filled material, Campanacci grade, and pathologic fracture, was not conducted to analyze the relevance between them and LC complex involvement, and the functional outcomes was not evaluated. Furthermore, it must be acknowledged that we only performed specific tumor ablation in the LC complex in limited cases with a short follow-up time. Therefore, we suggest that subchondral LC complex involvement and related surgical interventions should be evaluated using larger samples and more sophisticated methodologies, including controlled trials.

In conclusion, our discovery and characterization of the LC complex have contributed to improve anatomical and clinical understanding of knee joint and its related tumors. The LC complexes are anatomical structures that include the FTIE or IFF, cancellous bone canal surrounding blood vessel, and foramen-covered synovium. The discovery of LC complex refreshes the traditionally anatomical comprehension about the vasculature and canals in the epiphysis and metaphysis of distal femur and proximal tibia. Clinical data have confirmed its oncological significance in tumors around the knee. Based on its anatomical characteristics and the results of clinical investigations, LC complexes may facilitate the tumor recurrence and bidirectional tumor dissemination between intraosseous and intraarticular regions, therefore it may provide novel oncological viewpoints toward the surgery of knee-related tumors.

## Data Availability Statement

The raw data supporting the conclusions of this article will be made available by the authors, without undue reservation.

## Ethics Statement

The studies involving human participants were reviewed and approved by Medical Ethics Committee of Medical School Shandong University. The patients/participants provided their written informed consent to participate in this study.

## Author Contributions

JL, KC, and ZhenzL designed the study and supervised the investigation. XS and KC conducted the majority of this study and analyzed data. XS prepared the manuscript. TL, ZhenfL, QY, YL, and KL helped with correlative surgery. YY and XG helped with radiographic observations and measurements. FS, QL, and HH helped with histological and pathological examinations. AZ revised the manuscript. All authors contributed to the article and approved the submitted version.

## Funding

This research was supported by the National Natural Science Foundation of China (No.81672655), Qingdao Key Health Discipline Development Fund, and Scientific Research Foundation of Qilu Hospital of Shandong University (Qingdao) (No.QDKY2019YZ01).

## Conflict of Interest

The authors declare that the research was conducted in the absence of any commercial or financial relationships that could be construed as a potential conflict of interest.

## Publisher's Note

All claims expressed in this article are solely those of the authors and do not necessarily represent those of their affiliated organizations, or those of the publisher, the editors and the reviewers. Any product that may be evaluated in this article, or claim that may be made by its manufacturer, is not guaranteed or endorsed by the publisher.
