# Supplementary material for: Discovery of Lijianmin-Chengkun Complexes and Their Oncological Application in Osseous and Intraarticular Lesions Around the Knee
Source: Front Surg. 2021 Dec 3;8:691362. doi: 10.3389/fsurg.2021.691362 (PMC8677826; doi:10.3389/fsurg.2021.691362)
Supplement: Supplementary file 1 [file Data_Sheet_1.doc]

Supplementary Material

# Supplementary Methods

## Radiographic Observation and Measurement

Radiographic observations and measurements were performed using two CT scanners (Somatom Force and Philips Brilliance64-slice), and an MRI scanner (Magnetom Verio3.0T) with a dedicated 8-channel knee coil. From May 2017 to May 2020, the CT images (showed bilateral knee joints) from 128 adults and MRI or contrast-enhanced MRI images (showed unilateral knee joint) from 142 adults, from the Radiology Department of XX Hospitals were used for radiographic observation and measurement of FTIE and IFF. All of the radiographs were randomly selected following the inclusion and exclusion criteria and below.

The inclusion criteria for CT images: 1) Complete and clear CT images of the knee joint with axial scans using slice thickness of 1mm with no gap, reconstructed coronal, sagittal images, and qualified for 3D reconstruction. 2) The tibial intercondylar region and ICF are intact and clearly displayed on CT scans without obvious injury. 3) Age from 16-90 years old with a closed epiphyseal line.

The exclusion criteria for CT images: 1) The articular surface of the tibial plateau or ICF is severely damaged due to trauma, tumour, degenerative changes, acute or chronic inflammation and other factors. 2) The data of CT scan is incomplete or unavailable due to bad image storage.3) The patients subjected to surgery or invasive examination of the knee.

Following the above inclusion and exclusion criteria, 200 knees from 124 CT images were randomly selected for the study. The average age of the patients was 48.3±6.8 years old (16-88 years old). Among the 200 knees, 94 were left knees (47%) and 106 were right knees (53%). There were 141 healthy knees (70.5%), 29 knees (14.5%) with degeneration, 19 knees (9.5%) with trauma or sports injuries, and 11 knees (5.5%) with tumours.

The inclusion criteria for MRI: 1) Coronal, axial, and sagittal MRI scans with at least T1-weighted imaging (T1WI), turbo spin echo(TSE) fat-saturated T2-weighted imaging (T2WI) and TSE fat-suppressed T2WI sequences, slice thickness of 2 mm with no gap. 2) FTIE and the margins of tibia plateau, or IFF and the margins of ICF are clearly visible on the same image slice. 3) The tibial intercondylar region and ICF are intact and clearly displayed on MRI without obvious injury. 4) Age from 16-90 years old with a closed epiphyseal line.

The exclusion criteria for MRI: 1) MRI images with the FTIE and IFF not shown due to the slice thickness or other reasons. 2) The FTIE or IFF which could not be definitely confirmed due to inadequate imaging features. 3) Important anatomical structures such as ACL and PCL were severely damaged due to knee tumours, trauma, degenerative changes and other diseases. 4) The patients with surgery or invasive examination of the knee.

Following the above inclusion and exclusion criteria, 100 FTIEs and 225 IFFs shown on 142 MRI or contrast-enhanced MRI images were selected for the study. The MRI images are from 73 males and 69 females with averaged age of 35.2±6.3 years old (16-85 years old). They include 77 (54.2%) left knees and 65 (45.8%) right knees, 99 cases (69.7%) of sports injury, 28 cases (19.7%) of trauma, 10 cases (7.0%) of tumour and 5 cases (3.5%) of degeneration.

## Dissection Observation and Measurement

During May 2017-May 2020, 60 healthy adult knees were obtained from 24 cadavers and 16 patients with amputated lower extremities. Among them, 22 were males (55%) and 18 were females (45%), 28 were left knees (46.7%) and 32 were right knees (53.3%). The inclusion and exclusion criteria are displayed below.

The inclusion criteria: 1) Intact knee joint specimens obtained from patients with amputation due to car accidents or tumour, or from cadavers. 2) The femur and tibia are intact without obvious damage. 3) MGA and MGV are in good condition. 4) Adults of aged 16-90 years old with closed epiphyseal lines.

The exclusion criteria: 1) Important structures in the knee joint, especially the ICF and tibial intercondylar region are damaged by joint degeneration, injury, tumour, knee arthritis and other reasons. 2) History of knee surgery. 3) The cadaveric knee specimen is not well preserved, or the knee joint has been damaged in other anatomical studies.

Fourteen knees (7 left knees and 7 right knees) with tumour involvement (7osteosarcomas, 5 GCTBs, 1 chondrosarcomas and 1 diffused giant cell tumour of tendon sheath) were obtained from the patients(8 males and 6 females) who underwent segmental resection or amputation in our descriptive clinical study.

## Details for Geometric Measurement of location-indicating parameters

Since the shape of foramina is not a perfect circle the maximum diameter of a foramen of FTIE or IFF in axis, sagittal or coronal plane of CT images was measured.

The measurements for DA and DP are already described in Methods of the article and the definition of DP reduced the influence of posterior slope (Supplementary Fig.1A). Due to the FTIE is in the vicinity of tibial intercondylar eminence, we are unable to measure the DM and DL directly. In order to measure the DM and DL, we projected FTIE to the coronal baseline established by connecting medial and lateral margin of tibia plateau (Supplementary Fig.1B). Similarly, the IFF was projected on the line by connecting anterior and posterior margins of ICF if the IFF and two margins are not collineation (Supplementary Fig.1C, D). The principle for measurement of DM and DL was quite similar for tibia and femur (Supplementary Fig.1E). All the “margins” for measurement should be the furthest border of cartilage and they should only be marked in the same MRI layer as the foramen (mina) appeared.

**
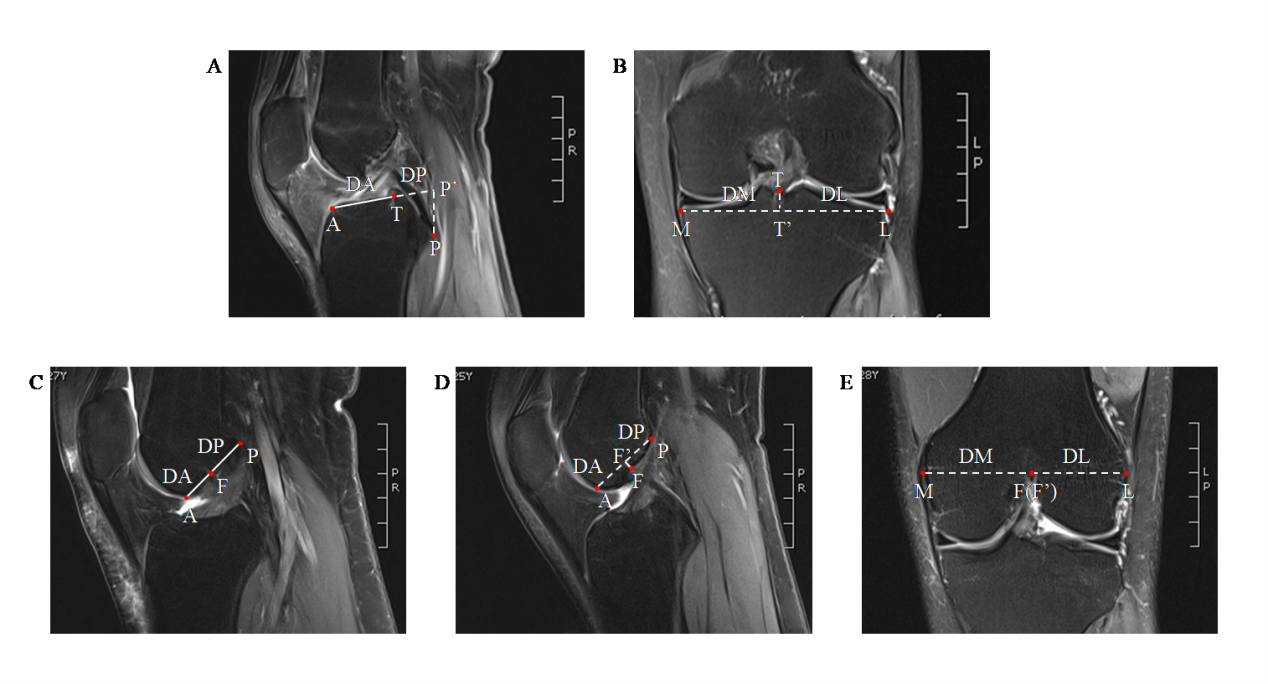
**

**Supplementary Figure 1.** Measurements of DA, DP, DM and DL of FTIE and IFF were demonstrated on MRIs. (**A**) The DA and DP in tibia were measured according to the method shown in image. T for the center of FTIE, A for the anterior margin of tibia plateau, P for the posterior margin of tibia plateau. P’ for the intersection point of DA extending line and the sagittal line which P lied on. (**B**) The DM and DL in tibia were measured according to the method shown in image. M for medial margin of tibia plateau, L for lateral margin of tibia plateau. T for the center of FTIE, T’ for the projection on the coronal baseline. (**C,D**) The DA and DP in femur were measured according to the method shown in image. F for the center of IFF, A for the anterior margin of ICF, P for the posterior margin of ICF, F’ for the projection on AP. (**C**) A, F and P points are collineation. (**D**) A, F and P points are not collineation. (**E**) The DM and DL in femur were measured according to the method shown in image. F for the center of IFF, M for the medial margin of ICF. L for the lateral margin of ICF. The F’ which is the projection of F would be used if necessary.

The measurements for DA and DP are already described in Methods of the article and the definition of DP reduced the influence of posterior slope (Supplementary Fig.1A). Due to the FTIE is in the vicinity of tibial intercondylar eminence, we are unable to measure the DM and DL directly. In order to measure the DM and DL, we projected FTIE to the coronal baseline established by connecting medial and lateral margin of tibia plateau (Supplementary Fig.1B). Similarly, the IFF was projected on the line by connecting anterior and posterior margins of ICF if the IFF and two margins are not collineation (Supplementary Fig.1C, D). The principle for measurement of DM and DL was quite similar for tibia and femur (Supplementary Fig.1E). All the “margins” for measurement should be the furthest border of cartilage and they should only be marked in the same MRI layer as the foramen (mina) appeared.

## Detailed Information for the Patients Enrolled in the Retrospective Clinical Study

The demographic data of the patients in the retrospective clinical study was listed in Supplementary Table 1.

**Supplementary Table 1** Patient demographics

| General information | Type A (n=8) | Type B (n=50) |
| --- | --- | --- |
| Mean age (sd) | 32.92 (10.65) | 36.39 (11.16) |
| Gender, n(%) |  |  |
| M | 6 (50.0%) | 36 (51.4%) |
| F | 6 (50.0%) | 34 (48.6%) |
| Location, n(%) |  |  |
| Femur | 7 (58.3%) | 43 (61.4%) |
| Tibia | 5 (41.7%) | 17 (24.3%) |
| Recurrence, n(%) | 6 (50.0%) | 17 (24.3%) |
| Time to recurrence(month, mean±SD) | 15.00±10.84 | 27.41±23.42 |

# Supplementary Results

## FTIE and IFF Are Surrounded by Accessory Foramina

In our radiographic observation, we found that both FTIE and IFF were surrounded by some accessory foramina. FTIE was accompanied by 1-3 satellite accessory foramina (maximum diameters ≤ 9mm) in about half of the individuals (Supplementary Fig.2A). In most situations, there existed 2-4 IFFs accompanied by 5-10 satellite accessory foramina. The accessory foramen is distinguished from IFF or FTIE mainly by the size, the depth of the connected bony canals and if it contains blood vessels. These accessory foramina were also observed in anatomical specimens (Supplementary Fig.2B).

**
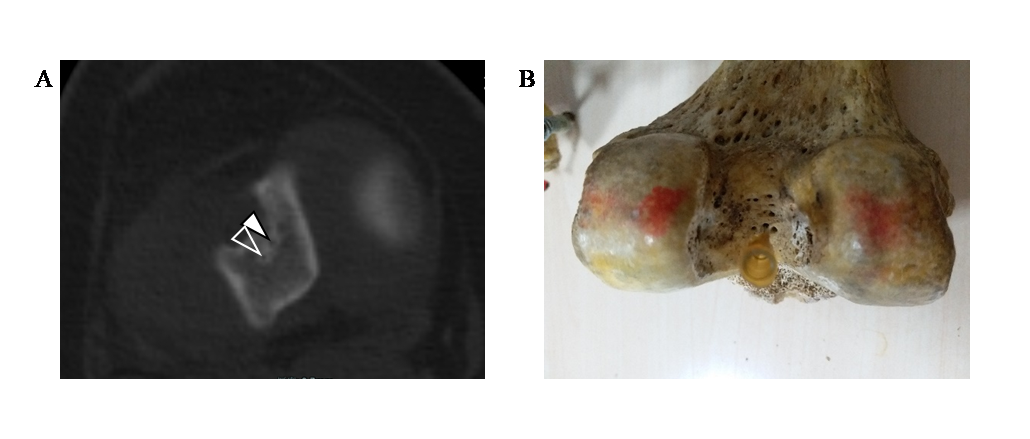
**

**Supplementary Figure 2.** Tibial and femoral accessory foramina were displayed. (**A**) Tibial accessory foramen(empty arrowhead) accompanied with the FTIE(arrowhead) on CT. (**B**) Femoral accessory foramina surrounded the IFFs in anatomical specimens.

## FTIE and IFF in Animals

The FTIE and IFF were also observed in a 10-month-old pig. There is a report of FTIE in rodents and macaques. Whether these foramina exist in other animals require further researches (Supplementary Fig.3).

**
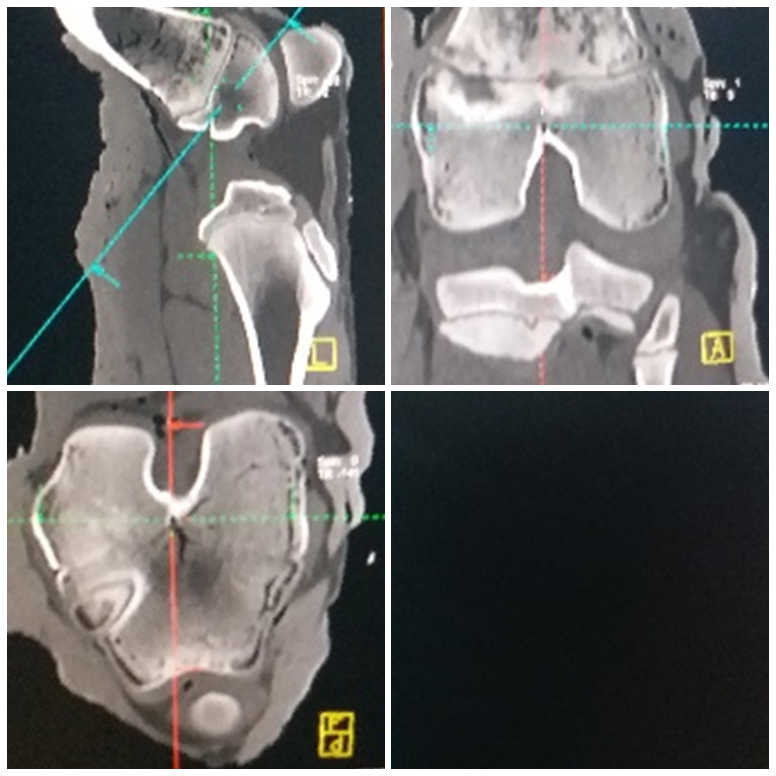
**

**Supplementary Figure 3.** IFF was observed in a 10-month pig.

## The Bony Canal Extended to the Diaphysis

The bony canal of LC complex may extend to the diaphysis of tibia in some specimens (Fig.4D). However it is not known where the LC complex is end with and whether it communicates with nutrient canal without further researches.

## Geometric Measurement between the foramina in LC complex and cruciate ligaments

A distance of 1.6mm to 12.3 mm between FTIE and tibial ACL insertion was determined in 88/100 cases (88%). In 12/100 cases, FTIEs (12%) located adjacent to the posterior edge of tibial ACL insertions, however, none of FTIEs surpassed the anterior edge of tibial ACL attachments. The distance between FTIE and tibial PCL insertion was determined to be 4.0 mm to 18.5 mm (Supplementary Fig.4). In most situations, we could not observe the femoral attachments of ACL and PCL on the same image slice which showed IFFs. In the images with visible IFFs, the rate of contemporaneous occurrence for femoral ACL and PCL insertions was 16.0% and 15.1%, respectively (Supplementary Fig.4).


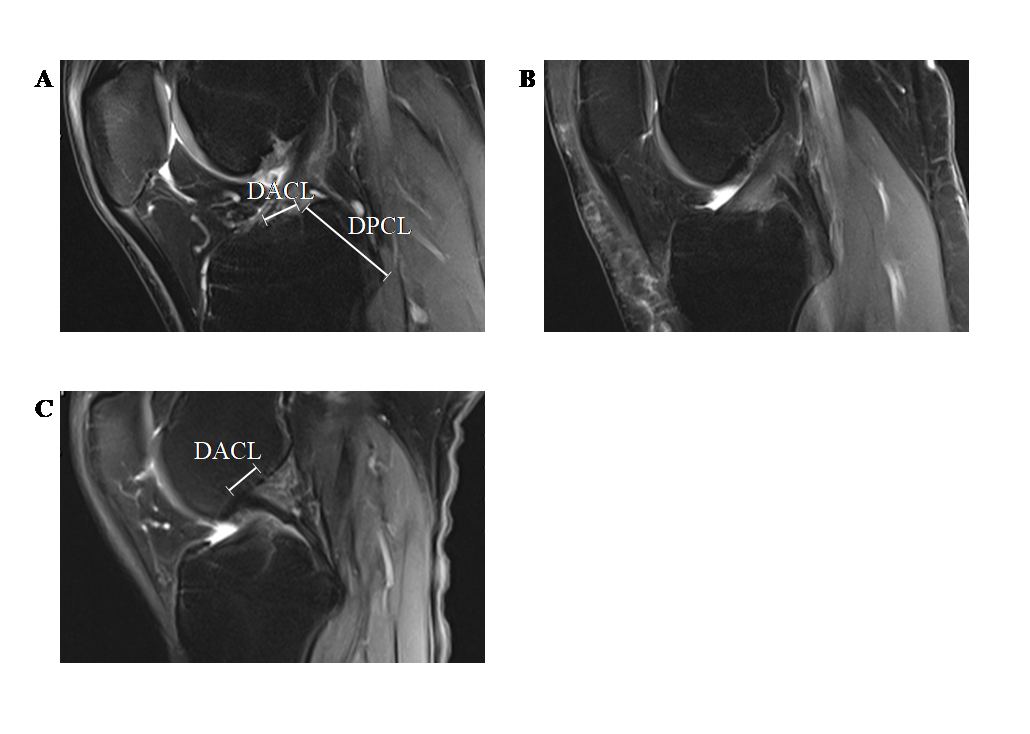


**Supplementary Figure 4.** The location of FTIE or IFF in relation to the tibial or femoral cruciate ligament insertions. (**A**) FTIE, the tibial insertions of ACL and PCL were shown on the same MRI slice. (**B**) Tibial and femoral insertions of ACL and PCL were not observed on MRI which showed the FTIE and IFF. (**C**) The femoral insertions of ACL and IFF were shown on the same MRI slice.

## Pathological Changes of Foramina-covered Synovium Under Arthroscopy Exploration

Pathological changes e.g. tumor infiltration and inflammation of foramina-covered synovium could be found not only in the anatomical specimen, but also under the arthroscopic exploration (Supplementary Fig.5). Pathological changes of foramina-covered synovium were observed in all the anatomical specimens and four arthroscopic exploration cases. It indicates that the foramina-covered synovium in LC complex may be sensitive to the aggressive or malignant tumor. So we also recommend to add intraoperative arthroscopic exploration in traditional surgery procedures when LC complex was involved. If pathological changes of foramina-covered synovium occurred, more radical operative plan should be considered.

**
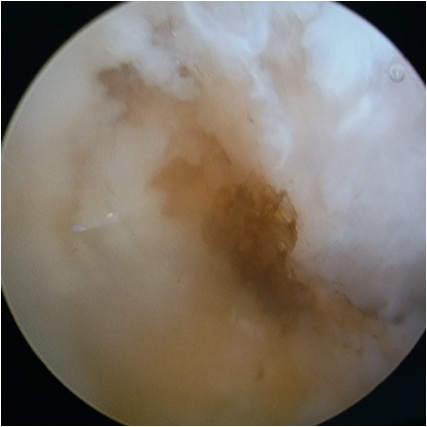
**

**Supplementary Figure 5.** The synovial degeneration was shown under arthroscopic observation.

## More Information for the Patients with Tumor Spread via LC Complex

For the patient (female, 19-year-old) whose images shown in Fig.5E-G, there was no obvious sign of recurrence after 32 months after we performed the tibial tumor segmental resection and prosthesis replacement (Supplementary Fig.6A). In addition, no recurrence was observed in another five GCTB patients who underwent segmental resection with a follow-up time of 5-30 months.

For the patient (female, 52-year-old) whose images shown in Fig.5J-L, the observation of intraarticular lesion on sagittal MRI shows that the tumor situates above the tibial intercondylar region (Supplementary Fig.6B). There was no obvious sign of recurrence at 28 months after we performed the intralesional curettage for the intraosseous tumor and LC complex and arthroscopic synovectomy for the intraarticular lesion.

**
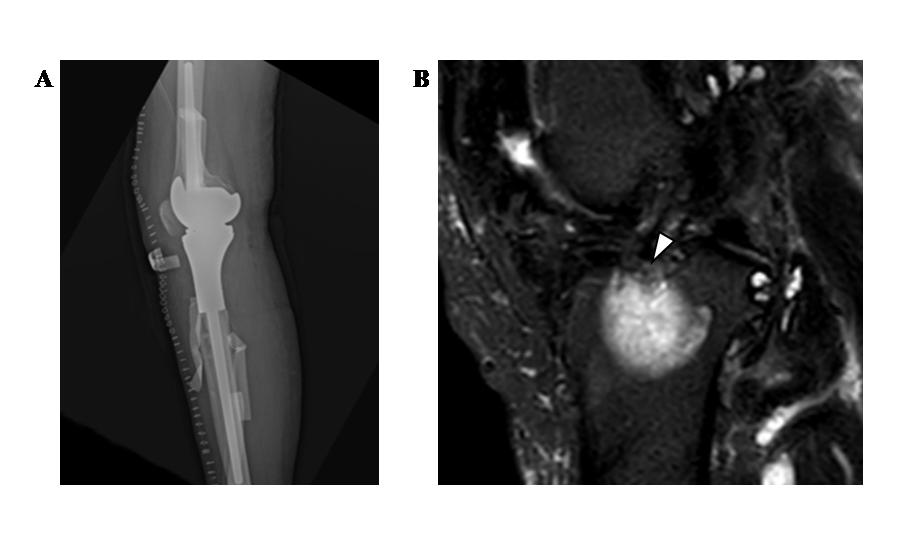
**

**Supplementary Figure 6.** More image information for the patients in the descriptive study was shown. (**A**) No local recurrence was observed in the postoperative radiograph after segmental resection for a 20-year-old female GCTB patient. (**B**) Intraarticular lesion (arrowhead) shown on MRI in a-52-year-old female with recurrent diffused giant cell tumor of tendon sheath.

## LC Complex Involvement in Metastatic Tumor

It is observed that the metastatic tumor spread from intraosseous to intraarticular regions via LC complex (Supplementary Fig.7A) in a 58-year-old female with pathological confirmed endometrial cancer (Supplementary Fig.7B).

**
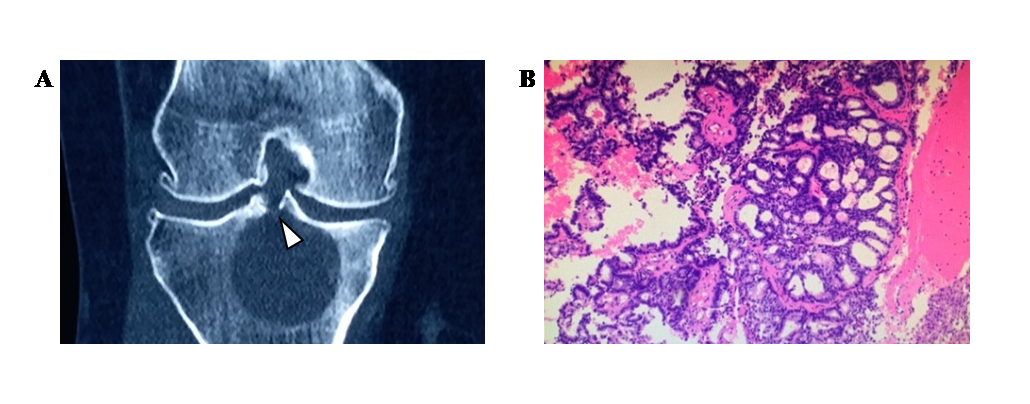
**

**Supplementary Figure 7.** Metastatic tumor spread from tibia to intraarticular regions via LC complex. (**A**) The intraosseous, intraarticular lesions and involved tibial LC complex were shown on CT coronal reconstruction. (**B**) Endometrial cancer was confirmed by the postoperative pathological examination.

## Tumor Invasion Affected the Diameter of LC Complex

The diameter of FTIE appears increased (Supplementary Fig.8A,B) when osteolytic tumor invaded into the LC complex. The IFF appears to be filled with tumor tissues particularly with invasive osteogenic tumors (Supplementary Fig.8C), so do the canals they connect to Fig.6B,E.

**
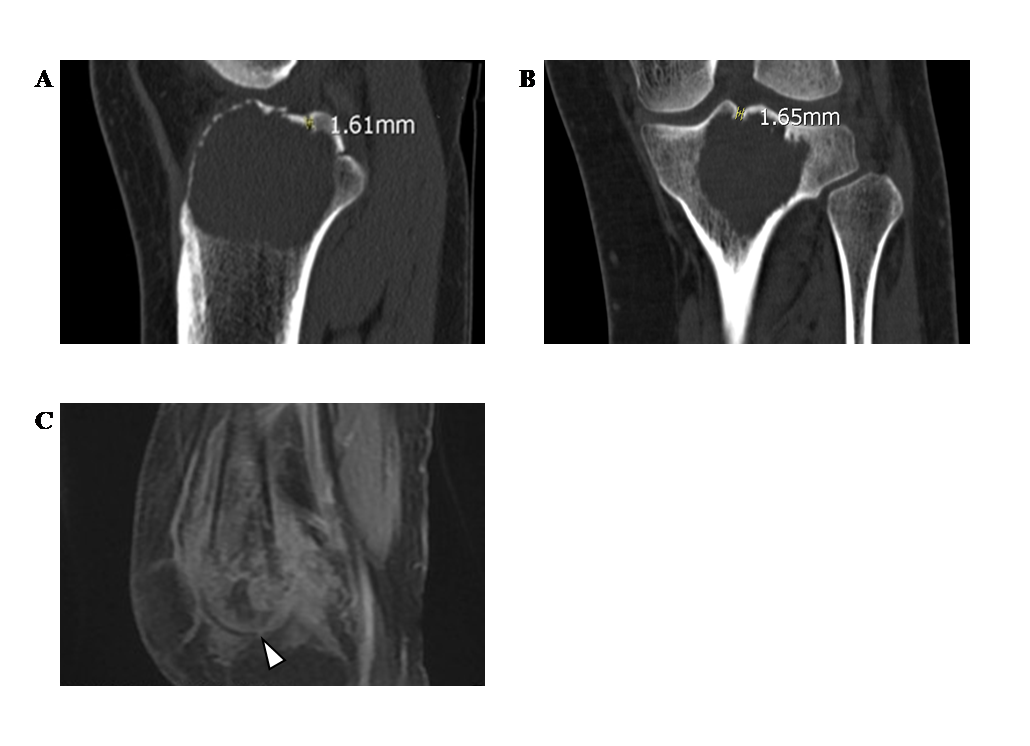
**

**Supplementary Figure 8.** Diameter varied when the tumor invaded the LC complex in some cases. (**A,B**) The diameter of FTIE expanded on CT sagittal and coronal reconstructions from a 20-year-old female whose tibial LC complex was involved in GCTB. (**C**) The IFF (arrowhead) was filled with osteosarcoma tissue from a 47-year-old female.

Therefore, we speculate that osteolytic lesions may cause enlarged diameters of LC complexes due to the bone destruction and osteogenic tumor cells accumulation in the canals so lead to narrow or invisible LC complexes.
